# Identifying brain systems for gaze orienting during reading: fMRI investigation of the Landolt paradigm

**DOI:** 10.3389/fnhum.2013.00384

**Published:** 2013-07-29

**Authors:** Rebekka Hillen, Thomas Günther, Claudia Kohlen, Cornelia Eckers, Muna van Ermingen-Marbach, Katharina Sass, Wolfgang Scharke, Josefine Vollmar, Ralph Radach, Stefan Heim

**Affiliations:** ^1^Department of Psychiatry, Psychotherapy and Psychosomatics, Medical School, RWTH Aachen UniversityAachen, Germany; ^2^Section Neurological Cognition Research, Department of Neurology, Medical School, RWTH Aachen UniversityAachen, Germany; ^3^Child Neuropsychology Section, Department of Child and Adolescent Psychiatry, Psychotherapy and Psychosomatics, Medical School, RWTH Aachen UniversityAachen, Germany; ^4^Department of Phoniatrics, Pedaudiology, and Communication Disorders, Medical School, RWTH Aachen UniversityAachen, Germany; ^5^Institute of Neuroscience and Medicine (INM-1), Research Centre JülichJülich, Germany; ^6^School of Psychology, The University of Queensland, St. LuciaBrisbane, QLD, Australia; ^7^General and Biological Psychology, Bergische Universität WuppertalWuppertal, Germany; ^8^Department of Psychology, Florida State UniversityTallahassee, FL, USA; ^9^JARA-Translational Brain MedicineAachen and Jülich, Germany

**Keywords:** reading, dyslexia, semantics, syntax, phonology, orthography, gaze, attention

## Abstract

The Landolt reading paradigm was created in order to dissociate effects of eye movements and attention from lexical, syntactic, and sub-lexical processing. While previous eye-tracking and behavioral findings support the usefulness of the paradigm, it remains to be shown that the paradigm actually relies on the brain networks for occulomotor control and attention, but not on systems for lexical/syntactic/orthographic processing. Here, 20 healthy volunteers underwent fMRI scanning while reading sentences (with syntax) or unconnected lists of written stimuli (no syntax) consisting of words (with semantics) or pseudowords (no semantics). In an additional “Landolt reading” condition, all letters were replaced by closed circles, which should be scanned for targets (Landolt's rings) in a reading-like fashion from left to right. A conjunction analysis of all five conditions revealed the visual scanning network which involved bilateral visual cortex, premotor cortex, and superior parietal cortex, but which did not include regions for semantics, syntax, or orthography. Contrasting the Landolt reading condition with all other regions revealed additional involvement of the right superior parietal cortex (areas 7A/7P/7PC) and postcentral gyrus (area 2) involved in deliberate gaze shifting. These neuroimaging findings demonstrate for the first time that the linguistic and orthographic brain network can be dissociated from a pure gaze-orienting network with the Landolt paradigm. Consequently, the Landolt paradigm may provide novel insights into the contributions of linguistic and non-linguistic factors on reading failure e.g., in developmental dyslexia.

## Introduction

**Figure 1:**
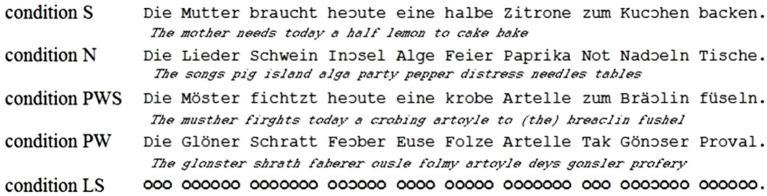
**Examples of the stimuli of all reading conditions**.

In reading, eye movements of children with developmental dyslexia differ from those of normal reading children (De Luca et al., 1999), e.g., more and longer fixations (Rayner, 1998; Hutzler and Wimmer, 2003). Although this fact has been known for more than 20 years, little is known about the causalities here: do these abnormal gaze patterns lead to dyslexic reading, or are they a consequence of reading difficulties potentially reflecting compensatory mechanisms? In order to address this question, a non-lexical and non-orthographical reading paradigm was developed (Corbic et al., 2007; Günther et al., 2012b; for the earlier work using the “Z-reading paradigm” see Ferretti et al., 2008). This “Landolt” paradigm allows investigating eye movements during reading without any influence of lexical information such as lexical frequency, phonotactics, or lexical status (Zschornak and Zeschmann, 2008; available at http://www.tguenthert.de/thesis/files/archive-2008.html; Zschornak et al., 2012; available at https://www.thieme-connect.de/ejournals/html/10.1055/s-0032-1304900). This non-lexical reading task only maintains the visual structure of written language, i.e., number of “letters” and “words.” This is achieved by replacing letters by non-orthorgraphic circle-like symbols, the so-called Landolt rings, thus removing all lexical, syntactic, or orthographic-phonological information. The visual structure is maintained by adopting all space characters. The size of the typefaces is matched exactly so that the length of a Landolt sentence is virtually identical to that of the matched (basic) sentence (see Figure 1). Hence, reading without words is stimulated, allowing to test whether the gaze patterns of the reader move over the Landolt sentences in a reading-like fashion.

Preliminary behavioral and eye-tracking data (e.g., Günther et al., 2012a,b; Radach et al., 2012 available at http://www.triplesr.org/conference/archive/2012/12conf-Abstracts.php)^1^ suggest that the Landolt paradigm mimics the spatial characteristics of eye movements during reading while without additional influence of lexical, syntactic, or orthographic-phonological sources. However, there is yet no neurophysiological evidence as to whether reading in the Landolt paradigm relies on the same brain regions relevant for orienting gaze during real reading or whether the seemingly comparable behavioral patterns of real reading and Landolt reading emerge from distinct neural mechanisms. Consequently, the aim of the present study was to characterize the neurofunctional network recruited for Landolt reading. We investigated whether or not the Landolt paradigm activates particular brain areas in addition to those supporting orthographic reading. Furthermore, in order to be able to dissociate these findings from brain areas involved in lexical, syntactic, and orthographic-phonological processing during reading (e.g., Vigneau et al., 2006, 2011; Price, 2010), we included additional conditions that would serves as functional localizers for each of these dimensions (Friederici et al., 2000a). Hence, we might be able to compare directly brain regions related to these dimensions to those involved in the Landolt paradigm, which supposedly relate to gaze orienting.

## Methods

### Participants

Twenty individuals (age range 20–30 years; mean 25;8 years; 10 women) participated in the study. All were right-handed according to the Edinburgh Inventory (Oldfield, 1971). They had normal or corrected-to-normal vision, normal reading skills (average percentile = 68) according to the Salzburger Lese- und Rechtschreibtest (SLRT-II; Moll and Landerl, 2010), a standard German reading test, and normal non-verbal intelligence (average IQ = 112; range = 90–130) in the revised version of the Cattell Culture Fair Text (CFT 20 R; Weiss, 2006).

### Materials and task

The study comprised five conditions (cf. Figure 1). Four of them contained orthographic stimuli with or without meaning (SEM+ vs. SEM−, i.e., words or pseudowords), which were arranged either as syntactically correct sentences (SYN+) or non-syntactic rows presented in a sentence-like fashion (SYN−). The first condition (sentences, S) was composed of 41 syntactic and semantic complete sentences. The material was taken from a study by Huestegge (2005). The next three conditions (pseudoword sentences, PWS; nouns, N; pseudowords, PW) were created according to the logic of the study by Friederici et al. (2000a). Pseudowords were created such that they were in accordance with the phonotactic and graphotactic rules of German. All stimuli were matched to the S condition with respect to the number of words, word length, number of syllables, syllable frequency, and German orthography. In order to ensure that all presented sentences began with an item from the same word class so that the initial landing position for each condition was not influenced by experimental manipulations, all sentences started with a definite determiner (der/die/das— “the”). Finally, for the fifth condition, all letters were replaced by Landolt rings not representing any orthographic, lexical, or syntactic information. These stimuli were constructed such that the “words” in the Landolt sentence (LS) were matched to all other conditions with respect to the number of “letters,” i.e., rings. Preliminary work showed that the eye movements for “reading” LS in search for targets were comparable to all other conditions (Kohlen, 2012; see also Hillen et al., 2012, available at https://www.thieme-connect.de/ejournals/html/10.1055/s-0032-1304899?update=true).

All stimuli were created such that they contained a varying number of open Landolt rings as targets. The number of targets was zero, one, or two. The positions of words or Landolt “words” containing targets were randomly distributed over the left, centre, and right part of the entire stimulus in order to prevent the subjects from engaging in processing strategies. The participants were asked to scan each stimulus in a reading-like fashion from left to right and to press the response button each time they detected a target. In analogy to the previous eye tracking studies (e.g., Zschornak and Zeschmann, 2008; Günther et al., 2013), each condition consisted of 18 sentences without targets, 14 sentences with one, and nine sentences with two targets. The total length of each stimulus varied from 55 to 68 characters, including all spaces, corresponding to a length of nine to twelve words per sentence. Landolt sentences and orthographic conditions were matched for visual angle covered and for number of characters.

In earlier studies using the Landolt paradigm (e.g.,Zschornak and Zeschmann, 2008; Günther et al., 2013), each letter was the size of 12 × 12 pixels, corresponding to the size of one Landolt ring. For the present study, the presentation of each stimulus had to be scaled down about two percent in order to make them fit the size of the screen used in the MRI scanner.

### Procedure

Before scanning, participants were screened for MRI suitability and were tested for reading ability, non-verbal intelligence, and handedness as outlined above. Right-handers with average or better reading and non-verbal IQ were included in the study. In preparation of scanning, the participants were informed about the procedures and the task. Next, they were familiarized with the upcoming stimuli. Finally, informed consent was obtained from all participants.

A very important aspect is the instruction researcher gives to participants. Using similar instructions for all conditions prevents subjects from engaging in different cognitive (and neurofunctional) processing modes. Kaakinen and Hyönä (2010) researched how gaze movement patterns of adult participants change according to the task instructions. They found that the gaze patterns of the participants behave very differently during proof reading than during reading comprehension tasks. Kaakinen and Hyönä (2010) suspected that the differences in gaze movement pattern are caused by different cognitive strategies. Therefore, in order to ensure comparable reading related eye movements in all conditions, the material of the present study contained targets in all reading conditions. As in the previous studies that used the Landolt paradigm, the participants were asked “to read the materials” and react whenever detecting a target. These targets look like left opened “c”s, i.e., they were no real orthographic signs but nonetheless highly comparable to those with respect to their visual features (see Figure 1).

In the scanner, stimuli were presented using the software Presentation 0.70 (Neurobehavioral Systems, San Francisco, CA, 2003) and an MR compatible goggle system (Resonance-Technologies) with a resolution of 800 × 600 pixels. Stimuli subtended a visual angle of 30 degrees. For each condition, 41 stimuli and seven null events were presented to the participants. Null events were included to improve modeling of the hemodynamic response function and to provide periods for a resting baseline. All conditions were divided into eight sub-blocks, each consisting of six trials (null events or stimulus trials). Generally the presentation sequence of the single stimuli and of the sub blocks was randomized. The distribution of the null events was pseudo-randomized for each block to ensure that no sub-block within this condition included more than two null events and that these events were separated by at least one real trial.

In accordance with data from a behavioral pilot study with 10 participants (5 males, aged 20–30 years), each experimental trial lasted for 4.5 s, including a 1-s blank screen between two stimuli. Sub-blocks were separated by periods of 4.5 s showing a blank screen. A total of 205 stimuli were presented with duration of 27 min for the fMRI session.

### Functional magnetic resonance imaging (fMRI)

Participants lay in a 3 Tesla magnetic resonance tomograph (Siemens TRIO) with standard head fixation using cushions. A total of 655 echo planar imaging (EPI) measurements were recorded from each subject. Whole-brain coverage was achieved by recording from 40 axial slices (interleaved acquisition) of 3 mm thickness (each with a distance of 1 mm), with a time of echo (TE) of 30 ms and with a time to repeat (TR) of 2500 ms. Thus, each voxel had a size of 4.0 × 4.0 × 3.0 mm^3^. The flip angle was 90°, the matrix 64 × 64 mm^2^, and the field of view (FOV) 200 mm. No subject showed head movements exceeding the size of one voxel so all participants remained in the analysis.

After the fMRI scans, an anatomical T1-weighted MP-RAGE sequence was run with a duration of 9 min (*TR* = 2500 ms; *TE* = 2.98 ms; 176 axial slices; FOV = 256 mm; flip angle = 9°).

### Data analysis

#### Behavioral data

Behavioral data were obtained from the individual Presentation log-files and analysed with SPSS 19.0 (SPSS Inc., 2011). By means of ANOVAs and subsequent pair-wise Bonferroni tests we analysed if the participants reacted equally correct to stimuli containing targets.

#### fMRI data

***Preprocessing.*** fMRI data were analysed with the computer software MATLAB 7.10.0 (The MathWorks, 2010) and SPM8 (The Welcome Trust Centre of Neuroimaging, 2009; http://www.fil.ion.ucl.ac.uk/spm/). The pre-processing took place in six steps. First, data were corrected for spatial movements during fMRI measurement (*realign*), followed by a temporal correction for acquisitions times (*slice time correction*). The normalization in the MNI reference space was accomplished using a *unified segmentation procedure* based on the individual anatomical scans. Prior to normalization, fMRI data were therefore realigned to this anatomical image (*coregister*). Finally, the data were smoothed with a Gaussian kernel of FWHM = 8 mm.

***Statistical analysis.*** At the first level data were analysed in a block design. The hemodynamic response function was modeled separately for the five conditions using the canonical HRF with the first time derivate. For each subject, five contrast images were generated by contrasting each condition against the implicit baseline condition.

During the second level analysis, contrast images of all 20 participants were entered into a flexible factorial repeated-measures design with the factors SUBJECT (as repetition factor) and CONDITION. The following contrasts were computed. First, activation for each condition was assessed relative to the implicit resting baseline. Next, a conjunction of these five contrasts was used to identify the network for gaze orientation involved in each condition. In order to determine brain regions specifically involved in the Landolt paradigm, the conjunction of the difference images of the Landolt condition minus each other condition [(LS − S) ∩ (LS − N) ∩ (LS − PWS) ∩ (LS−PW)] was computed.

In order to test whether processing in the Landolt paradigm involved lexical, syntactic, or orthographic processing, the following contrasts were calculated based on the study design. Regions involved in orthographic processing during reading were identified by the conjunction of the contrasts of all orthographic conditions against the Landolt condition [(S − LS) ∩ (N − LS) ∩ (PWS − LS) ∩ (PW − LS)]. Lexical processing was operationalized by contrasting conditions including words against those involving pseudowords (S + N − PWS − PW). Similarly, regions for syntactic processing were identified by contrasting sentences and pseudoword sentences against lists of words or pseudowords (S − N + PWS − PW).

All contrasts were assessed at a significance level of *p* < 0.05 FWE corrected at cluster level, obtained by using an uncorrected threshold of *p* ≤ 0.001 with a minimal cluster size (*k*) of 200 voxels.

***Neuroanatomical localization.*** For the precise neuroanatomical localization of the effects, we used the Jülich-Düsseldorf probabilistic atlas, which is based on an observer-independent analysis of cytoarchitectonic borders in a sample of ten post-mortem brains (Zilles et al., 2002; Schleicher et al., 2005). The atlas provides information about the position and variability of cortical regions in standard MNI reference space. For the assignment to cytoarchitectonically defined regions we used the SPM Anatomy Toolbox (Eickhoff et al., 2005) available at http://www.fz-juelich.de/inm/inm-1/DE/Forschung/_docs/SPMAnatomyToolbox/SPMAnatomyToolbox_node.html.

## Results

### Behavioral data

Participants pressed a response button whenever they thought there was a target within the stimulus. The data were obtained from all 205 items, divided into five conditions (S, N, PWS, PW, LS). Each condition block comprised 41 stimuli with either no, one, or two targets. Because of technical difficulties one data set was incomplete, lacking responses for 12 items (six items in conditions S and N, respectively). The descriptive data are presented in Table 1.

**Table 1:** **Behavioral data as a function of condition**.

**Condition**	**Hits**	***SD***	**Misses**	***SD***	**False Alarms**	***SD***
S	36.6	2.9	1.6	1.7	2.6	1.3
N	32.4	4.6	5.3	4.6	2.8	1.3
PWS	31.2	4.1	5.9	2.9	4.0	3.1
PWR	30.1	5.5	8.2	4.7	2.8	2.6
LS	29.3	5.5	10.2	5.7	1.5	1.5

The maximum of hits was 41 per condition. For each condition, the number of hits, misses, and false alarms is indicated together with the standard deviation.

S, sentences; N, nouns; PWS, pseudoword sentences; PWR, pseudoword rows; LS, Landolt sentences.

#### Accuracy per condition

The influence of experimental condition on the number of hits was tested in a one-way ANOVA with CONDITION (S, PWS, N, PW, LS) as factor. There was a main effect of CONDITION [*F*_(4, 19)_ = 7.664; *p* < 0.001]. *Post-hoc* Bonferroni tests revealed that condition S showed significantly more correct responses in comparison to condition PWS (*p* = 0.004), condition PW (*p* < 0.001), and LS (*p* < 0.001). Moreover, there was a trend toward more correct responses compared to condition N (*p* = 0.054). The conditions N, PWS, and PW did not differ with respect to correct responses (each *p* > 0.999). Likewise, during Landolt reading, performance was comparable in comparison with these other conditions (N: *p* = 0.362; PWS: *p* > 0.999; PW: *p* > 0.999).

#### Detailed error analysis: misses vs. false alarms

Overall, participants performed correctly on 78% of all trials. In the remaining 22% incorrect responses participants missed a target in 15% of the cases or produced false alarms in 7%.

Analysing these response patterns separately for each condition in a series of one-way repeated-measures ANOVAs revealed a main effect of RESPONSE TYPE (hit/miss/false alarm) in all conditions [S: *F*_(2, 19)_ = 1861.957; *p* < 0.001; N: *F*_(2, 19)_ = 372.177; *p* < 0.001; PWS: *F*_(2, 19)_ = 396.794; *p* < 0.001, PW: *F*_(2, 19)_ = 213.197; *p* < 0.001; LS: *F*_(2, 19)_ = 186.079; *p* < 0.001]. The *post-hoc* Bonferroni tests revealed no differences between misses and false alarms for S blocks (*p* = 0.453), W blocks (*p* = 0.128), and PWS blocks (*p* = 0.274). In contrast, for PW and LS blocks, there were differences between the types of incorrect responses (PW: *p* = 0.001; LS: *p* < 0.001). For both conditions, participants made more misses than false alarms.

### fMRI data

In this section, the fMRI data are reported with respect to the macroanatomical structures, which were activated in each condition; for detailed information about the cytoarchitectonic localizations please refer to the figures and tables. Results are reported separately for each condition contrasted against the resting baseline and for contrasts representing the gaze orientation network, orthographic processing, processing in the Landolt paradigm, semantic processing, and syntactic processing.

**Figure 2:**
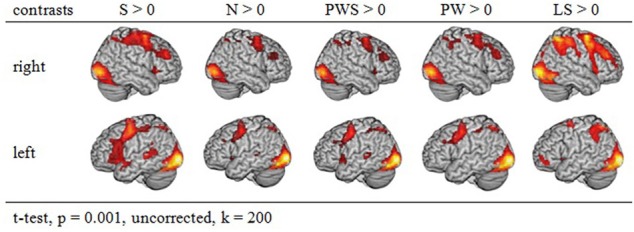
**Activation of similar reading conditions in contrast to the baseline**.

Figure 2 gives an overview of the brain activation for each condition compared to the resting baseline. In all conditions, there was comparable activation in the visual cortex, precentral gyrus, and right and left parietal lobe. Moreover, all conditions involving real letters (S, PWS, N, PW) showed activation in the left fusiform gyrus. In contrast, this region was not involved when reading the Landolt sentences.

#### Common gazing network

**Figure 3:**
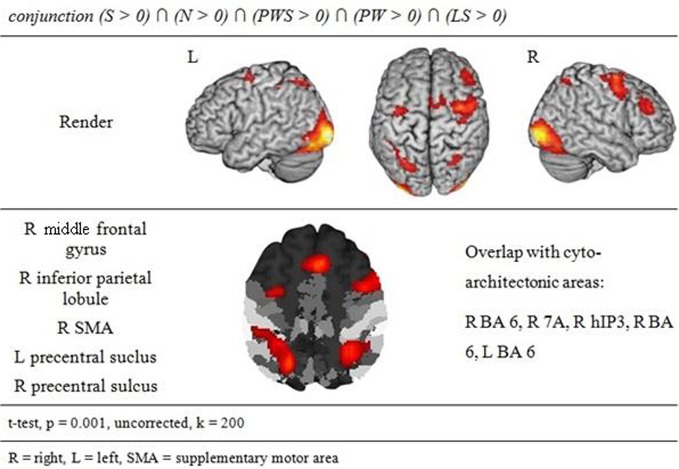
**Common gazing network**.

The conjunction analysis to identify a common gaze network of all reading conditions shows a brain activation pattern which is responsible for gaze patterns independent of activation related to semantic, syntactic, or orthographic processing (see Figure 3 and Table 2). Both hemispheres showed comparable patterns of activation. The largest cluster was located in the occipital lobe, with its local maximum in the cerebellum. The most anterior border of that cluster in the left ventral occipito-temporal cortex was in the fusiform and inferior temporal gyri (*y* = −56); the cluster extended posteriorly into the visual cortex (*y* = −105). Further clusters were found in the right and left middle frontal gyrus (MFG) extending into the superior frontal (SFG) and precentral gyri (PrCG), right inferior parietal lobule (IPL) extending into the right intraparietal sulcus (IPS) and right superior parietal lobule (SPL), and in the left caudate nucleus and putamen.

**Table 2:** **Common gazing network**.

**Conjunction (S > 0) ∩(N > 0) ∩ (PWS > 0) ∩ (PW > 0) ∩ (LS > 0)**							
**Cluster (size)**	**Local maximum in macro anat. structure**	**MNI**			***T*_max_**	**Overlap of cluster with cyto-architectonic area (in %)**	**Partial overlap of clusters with macro anat. structures**
		***x***	***y***	***z***			
1 (11850 voxels)	L collateral gyrus	−32	−98	−6	14.41	6.5% R area 17^1^	R LG
							L IOG
							R IOG
							L pFG
							Cerebellar
							Vermis
							L LG
							L CS
2 (1162 voxels)	R middle frontal gyrus	34	0	66	8.00	17.1% R area 6^2^	R SFG
							R PrCG
3 (1084 voxels)	R inferior parietal lobule	30	−52	46	6.54	24.4% R 7A^3^	R SPL
						20.4% R hIP3^3^	R IPS
4 (881 voxels)	R SMA	2	14	52	8.71	31.9% R area 6	R SFG
						13.2% L BA6	L PrCG
5 (613 voxels)	R precentral sulcus	38	38	24	6.04		R MFG
6 (485 voxels)	L precentral sulcus	−30	−4	52	5.86	13.7% L area 6	L PrCG
							L SFG
							L MFG
7 (277 voxels)	L Nucleus caudatus	−18	−10	22	5.68		L Putamen

t-test, p < 0.001, uncorrected, k = 200.

R, right; L, left; LG, lingual gyrus; IOG, inferior occipital gyrus; pFG, posterior fusiform gyrus; CS, calcarin sulcus; SFG, superior frontal gyrus; PrCG, precentral gyrus; SPL, superior parietal lobe; IPS, intraparietal sulcus; MFG, middle frontal gyrus; SPL, superior parietal lobe Lobulus; SMA, supplementary motor area; PrCG, precentral gyrus.

1 Amunts et al. (2000)

2 Geyer (2003)

3 Scheperjans et al. (2008).

#### Orthographic processing

**Figure 4:**
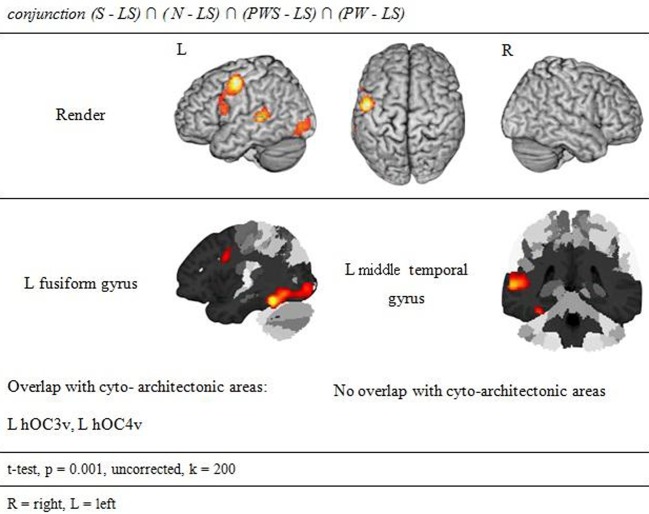
**Orthographic Processing**.

Figure 4 and Table 3 show the brain activation patterns for orthographic processing. Except for the Landolt paradigm, all other conditions required orthographic processing. Consequently, the conjunction analysis of the differences between each single orthographic condition (S, N, PWS, PW) and the Landolt condition reveals brain areas relevant for the processing of orthographic information. This conjunction analysis of the processing of orthographic information shows a clear difference in hemispheres, with activation predominantly in the left hemisphere. The biggest cluster was in the left fusiform gyrus, starting anteriorly at *y* = −40 and extending posteriorly into the ventral visual cortex (y = −98). Another cluster was located in the left PrCG, reaching further anterior into the left inferior frontal gyrus (IFG). In the right hemisphere, activation was found in the cerebellum, PrCG, and middle temporal gyrus (MTG).

**Table 3:** **Orthographic processing**.

**conjunction (S − LS) ∩ (N − LS) ∩ (PWS − LS) ∩ (PW − LS)**							
**Cluster (size)**	**Local maximum in macro anat. structure**	**MNI**			***T*_max_**	**Overlap of cluster with cyto-architectonic area (in %)**	**Partial overlap of clusters with macro anat. structures**
		***x***	***y***	***z***			
1 (1625 voxels)	L fusiform gyrus	−38	−48	−20	7.09	13.4% L hOC3v^4^	L MOG
						9.2% L hOC4v^4^	L IOG
2 (1510 voxels)	L precentral gyrus	−50	−6	50	8.29	34.2% L area 44^5^	L IFG
						20.7% L area 6	
3 (661 voxels)	L middle temporal gyrus	−64	−36	4	7.10		
4 (352 voxels)	R Cerebellum	24	−64	−50	7.26	22.2% R Lobule VIIa^6^	
						16.9% R Lobule VIIa	
						Crus II^6^	
						14.4% R Lobule VIIa^6^	
5 (318 voxels)	R Cerebellum	22	−64	−24	6.18	99.4% R Lobule VI^6^	

t-test, p < 0.001, uncorrected, k = 200.

R, right; L, left; MOG, middle occipital gyrus; IOG, inferior occipital gyrus; IFG, inferior frontal gyrus.

4 Rottschy et al. (2007)

5 Amunts et al. (1999)

6 Diedrichsen et al. (2009).

#### Processing in the Landolt paradigm

**Figure 5:**
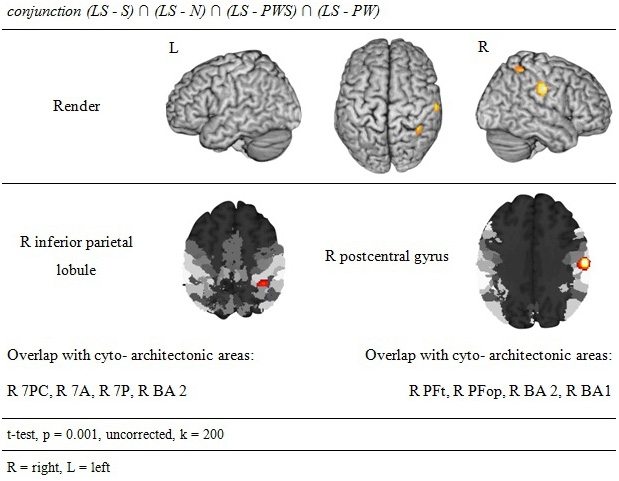
**Processing in the Landolt paradigm**.

The reverse analysis shows brain areas with greater activation in the Landolt paradigm than in all other conditions, which contained orthographic stimuli (Figure 5 and Table 4). Two clusters were observed. The maximum of the first clusters was localized in the right IPL, extending into the precuneus and the SPL. The local maximum of the second cluster was localized in the right postcentral gyrus (PoCG). Plotting the beta estimates of the activation strength revealed uniformly strong effects for LS in comparison to all other conditions in comparison to lower positive signal in the IPL vs. negative signal in the PoCG (Figure 8).

**Table 4:** **Processing in the Landolt paradigm**.

**conjunction (LS − S) ∩ (LS − N) ∩ (LS − PWS)∩ (LS − PW)**							
**Cluster (size)**	**Local maximum in macro anat. structure**	**MNI**			***T*_max_**	**Overlap of cluster with cyto-architectonic area (in %)**	**Partial overlap of clusters with macro anat. structures**
		***x***	***y***	***z***			
1 (402 voxels)	R inferior parietal lobule	38	−46	54	4.85	29.1% R 7PC^3^	R Prenuceus
						21.9% R 7A	R SPL
						16.9% R 7P^3^	
						15.6% R area 2^7^	
						9.5% R hIP3^3^	
2 (348 voxels)	R postcentral gyrus	62	−18	32	6.08	42.0% R PFt^8^	R IPL
						17.8% R PFop^8^	
						14.7% R area 2	
						10.8% R area 1^9^	
						10.5% R area 3b^9^	

t-test, p < 0.001, uncorrected, k = 200.

R, right; L, left; SPL, superior parietal lobule; IPL, inferior parietal lobule.

3 Scheperjans et al. (2008)

7 Grefkes et al. (2001)

8 Caspers et al. (2006)

9 Geyer et al. (1999), (2000).

#### Semantic processing

**Figure 6:**
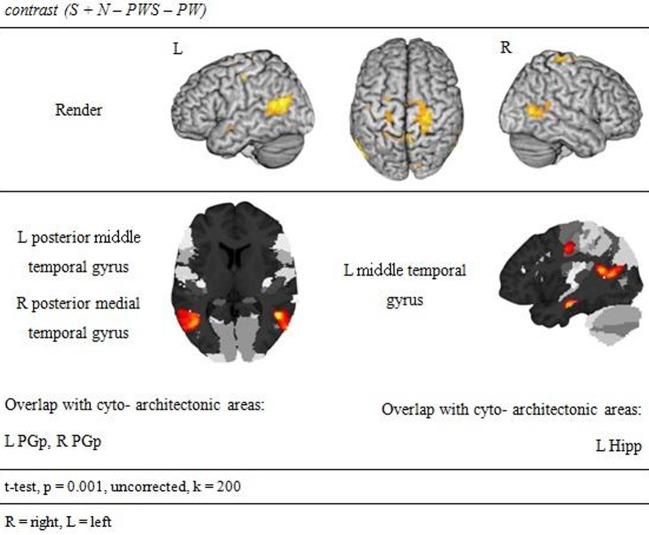
**Semantic processing**.

Subtracting activation for pseudoword lists and sentences from activation for reading real-word sentences and nouns identified regions involved in semantic processing during reading. This semantic contrast showed activation predominantly in temporal and parietal areas of both hemispheres. Bilaterally, the angular gyri of the IPL, PrCG, MTG, and precuneus were activated. Moreover, activation extended into the right cuneus (Figure 6 and Table 5).

**Table 5:** **Semantic processing contrast**.

**(S + N − PWS − PW)**							
**Cluster (size)**	**Local maximum in macro anat. structure**	**MNI**			***T*_max_**	**Overlap of cluster with zyto-architekt. Area (in %)**	**Partial overlap of clusters with macro anat. structures**
		***x***	***y***	***z***			
1 (3053 voxels)	R precentral gyrus	24	−34	66	5.50	21.0% R area 6	R Prenuceus
						15.0% L area 6	L MCC
						8.4% L area 4p^10^	L GPoC
						6.0% L 5M^3^	R MCC
						5.7% R area 4p	L Prenuceus
							R SMA
							L SMA
							L SPL
2 (1292 voxels)	L posterior middle temporal gyrus	−46	−62	12	5.72	13.1% L PGp^8^	L IPL
							L GA
3 (633 voxels)	R posterior middle temporal gyrus	48	−52	8	5.11	5.9% R PGp	R GTS
							R GA
							R STS
4 (322 voxels)	L middle temporal gyrus	−40	−14	−20	4.72	6.1% L Hipp^11^	L Hippo-campus
5 (309 voxels)	R Cuneus	10	−86	24	3.84	22.0% R area 18^1^	R GOS

t-test, p < 0.001, uncorrected, k = 200.

R, right; L, left; MCC, middle cingulate cortex; PrCG, precentral gyrus; GPoC, postcentral gyrus; SMA, supplementair motor area; SPL, superior parietal lobe; IPL, inferior parietal lobe; GA, angular gyrus; GTS, superior temporal gyrus; STS, superior temporal sulcus; GOS, superior occipital gyrus.

1 Amunts et al. (2000),

3 Scheperjans et al. (2008),

8 Caspers et al. (2008),

11 Amunts et al. (2005).

#### Syntactic processing

**Figure 7:**
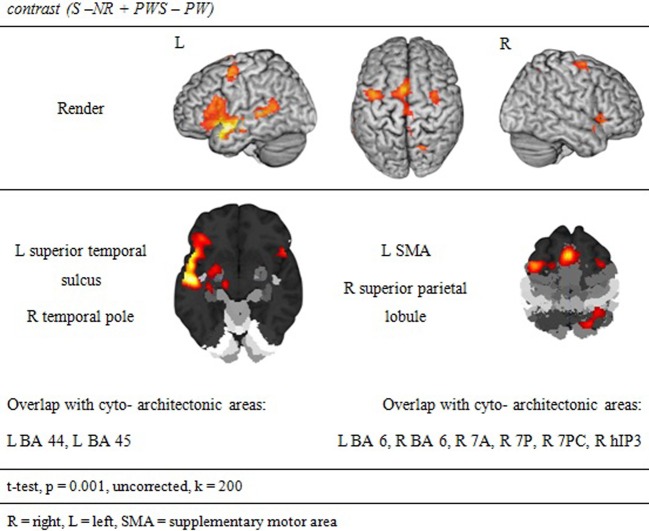
**Synaptic processing**.

**Figure 8:**
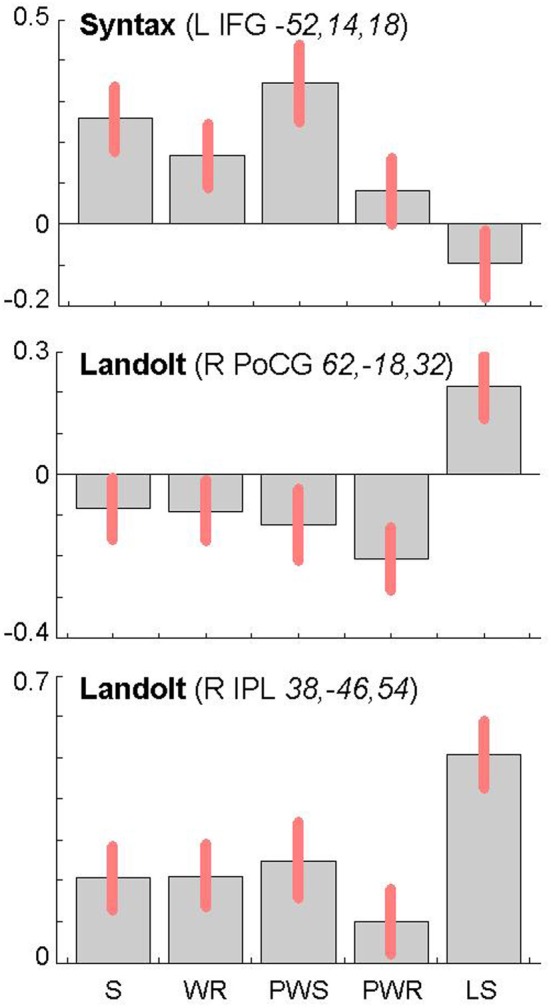
**Contrast estimates and 90% confidence intervals for main local maxima in the left inferior frontal gyrus (top) and the right parietal lobe (middle and bottom) as a function of experimental condition (S, sentences; WR, rows of words; PWS, pseudoword sentences; PWR, rows of pseudowords; LS, Landolt sentence)**. The MNI coordinates (*x*, y, z) of the local maxima from which the estimates were derived are provided together with the name of the corresponding contrast.

Syntactic processing was reflected in the contrasts of sentences (containing real words, S, or pseudowords, PWS) minus lists of nouns or pseudowords (N, PW). These findings are reported in Figure 7 and Table 6. The results show an explicit hemispheric distinction: effects in the left hemisphere and in particular in the left frontal lobe were stronger than in the right hemisphere. The biggest cluster was localized in the left fronto-temporal region, covering the superior temporal sulcus (STS), superior temporal gyrus (STG) and MTG as well as left IFG and insula. The second cluster was found in the left SMA, extending into left SFG. In the right hemisphere, a smaller fronto-temporal cluster covered the temporal pole, IFG, and insula. Further effects were found in the right PrCG and MFG, SPL, and cerebellum. In order to compare these data to earlier findings from the auditory modality by Friederici et al. (2000a), we checked whether the effect in Broca's region was due to higher activation for both real sentences and pseudoword sentences. This was indeed the case (cf. Figure 8).

**Table 6:** **Syntactic processing**.

**contrast (S −N + PWS − PW)**							
**Cluster (size)**	**Local maximum in macro anat. structure**	**MNI**			***T*_max_**	**Overlap of cluster with zyto-architekt. Area (in %)**	**Partial overlap of clusters with macro anat. structures**
		***x***	***y***	***z***			
1 (6299 voxels)	L superior temporal sulcus	−54	2	−14	8.32	8.6% L area 44	L STG
							L MTG
							L PT
							L Insula
							L IFG
2 (2459 voxels)	L SMA	−2	10	54	6.94	41.2% L area 6	L PrCG
						14.5% R area 6	R MCC
							L SFG
3 (573 voxels)	R temporal pole	52	16	−14	4.85	15.2% R area 45^5^	R Insula
							R IFG
							R Putamen
4 (323 voxels)	R superior parietal lobulus	22	−66	56	4.96	50.5% R 7A	R SPL
						25.3% R 7P	
						14.4% R 7PC	
						8.3% R hIP3	
5 (264 voxels)	R cerebellum	16	−66	28	5.46	48.1% R Lobulus VI^6^	Cerebeallar vermis
						19.9% R Lobulus^6^	
						9.6% L Lobulus VI^6^	
						5.1% R Lobulus VIIa Crus II	
6 (203 voxels)	R superior frontal gyrus	34	−2	66	4.43	6.9% R area 6	R MFG
							R PrCG

t-test, p < 0.001, uncorrected, k = 200.

R, right; L, left; MTG, middle temporal gyrus; PT, temporal pole; IFG, inferior frontal gyrus; SPL, superior parietal lobe; PrCG, precentral gyrus; MCC, middle cingulate cortex; SFG, superior frontal gyrus; PrCG, precentral gyrus.

5 Amunts et al. (1999)

6 Diedrichsen et al. (2009).

## Discussion

The present study aimed to identify the neural systems that support reading-like behavior in the novel Landolt reading task, a paradigm developed to study eye movements during reading without influences of lexical, syntactic, or phonologic-orthographic processing. The results of the present study allow a description of the neuronal processing during reading in the Landolt paradigm, dissociating them from those regions relevant for the linguistic dimensions. The main finding was that the Landolt paradigm and the other reading conditions activated a common network relevant for gaze orienting.

### Common gazing network

The conjunction analysis of all five conditions showed brain activation of a common gazing network, which is necessary for reading independent of language processing. Besides prominent activity in the left and right visual cortex (including cytoarchitectonic areas 17 and 18; Amunts et al., 2000), there were significant activations around the middle frontal gyrus in both hemispheres. These clusters extended posteriorly into area 6 in the SMA and PrCG (Geyer, 2003), a region regulating gaze orienting during saccadic movements (Grosbras et al., 1999; Haller et al., 2008).

In parietal lobe, the gazing network included an area around the right inferior parietal lobule extending into cytoarchitectonic area hIP3 in the intraparietal sulcus (Scheperjans et al., 2008). Fan et al. (2005) discuss both IPL and IPS in relation to attention regulation networks, especially in the alerting and the orienting network. Barthélémy and Boulinguez (2002) and Culham et al. (2006) reported that the right inferior parietal lobule is involved in attention processes and in the neuronal planning of movements and the visuo-motor conversion. Related to this, Thiel et al. (2003) described that this area was involved in visual selection tasks. Taken together, these findings suggest that gaze re-orienting has an attentional and in particular a visuo-spatial component. Thiel et al. (2003) and Fan et al. (2005) define the alerting network as a network that is activated while waiting and expecting a following target. It is possible that this expecting attitude is common with the position of the participants in the actual study. Both aspects, i.e., attention (Thiel et al., 2003) and the planning and conversion of gaze movements (Barthélémy and Boulinguez, 2002) are likely to contribute to the activation in this area in the present study. This point will become relevant later when discussing the activation patterns specific for the Landolt paradigm.

### Orthographic processing

The presence or absence of orthographic/phonologic information is the most important difference between the orthographic (S, N, PWS and PW) and the Landolt (LS) reading conditions. Consequently, contrasting orthographic and non-orthographic conditions yields a pinpointed neurophysiological description of the Landolt paradigm. The conjunction analysis for orthographic processing shows a clear difference in the hemispheres. Right-hemispheric activations were localized in the cerebellum, while left-hemispheric clusters were found in the fusiform, precentral, and middle temporal gyrus. The fusiform gyrus as part of the ventral stream has repeatedly been shown to support the processing of written stimuli. This observation has led to the idea of the visual word form area (e.g., Dehaene et al., 2002, 2010) which is, however, not undisputed (see also Price and Devlin, 2003, 2011; Price, 2012; for comprehensive accounts). The fact that reading orthographic stimuli involved the fusiform gyrus to a significantly higher degree than the Landolt sentences might thus be taken to suggest that the Landolt stimuli were indeed not processed in an orthographic way (e.g., modulated by top-down expectancy).

Further left temporal activations for orthographic processing were found in the left middle temporal gyrus in the vicinity of Wernicke's area. Jobard et al. (2003) reported that this area is involved while reading words and pseudowords. They proposed that grapheme-to-phoneme conversion is represented here and in the superiorly adjacent superior temporal gyrus and sulcus.

Finally, there was also cerebellar activation for orthographic processing. Originally, activations in the cerebellum had been expected for the control of gaze movements (Kheradmand and Zee, 2011) rather than orthographic processing. Interestingly, however, there were only few activations at all in the common gazing network, but rather more prominent effects for orthographic processing. These data are interesting in the context of the cerebellar hypothesis of dyslexia (e.g., Nicolson et al., 1999) that assumes that general problems of automaticity affect successful reading in developmental reading disorders. These, in turn, relate to reduced cerebellar activation and likewise to reduced cerebellar volumes (Pernet et al., 2009). The present data suggest that this failure might not be localized (exclusively) at a general procedural level but might pertain in particular to fine-grained visual information such as letters.

### Specifics of the Landolt paradigm

The conjunction analysis to detect the neuronal activation for reading Landolt sentences in comparison to orthographic material revealed two activation clusters in the right hemisphere in the inferior parietal lobule and precentral gyrus. This finding stands in contradiction to the initial hypothesis that Landolt reading does not activate any regions to a higher degree than “real” orthographic reading.

This pattern of activations resembles at least partly the pattern found for gaze orienting discussed above. Like for gaze orienting, the right inferior parietal lobule was involved in Landolt reading. This finding suggests that Landolt reading in fact requires visual-spatial attention even to a higher degree than normal reading, probably because parafoveal vision does not help identifying appropriate landing positions for subsequent saccades (but note that the landing positions *are* indeed appropriately identified, but at the expense of longer re-fixation times; cf. Günther et al., 2012a,b). The present neuroimaging results indicate that this performance was achieved by stronger involvement of the right parietal attention and gaze orienting network. The mechanism behind this recruitment might be found in the automaticity of the normal reading process in adult readers, which is not exactly given in the new context of “reading” lines of circles and deliberately looking for targets—perhaps even more so in the Landolt condition where all stimuli almost look alike.

The second cluster was located in the somatosensory cortex in the postcentral gyrus (Geyer et al., 1999; Grefkes et al., 2001). Earlier studies on visual processing (e.g., Donner et al., 2000) have reported networks in healthy controls that contain exactly these two regions, showing that this network is indeed relevant for covert visual selection—a process necessary in the Landolt task to plan the next saccade (please also see the “Limitations” section below). More recent work by Balslev et al. (2012) refined these data, showing that under specifically demanding conditions eye proprioception may be used to guide gaze behavior, whereas normally no such feedback information is necessary. In the Landolt paradigm, the fact that the “letters,” i.e., the rings, all look alike may have led the brain to rely additionally on proprioceptive information to perform a visual gaze pattern that is comparable to that in normal reading. We say “additionally,” because it is likely that the task to detect a target in *all* conditions would involve a certain degree of visual search also in the orthographic conditions (even thought the beta estimates of the activation were negative for the other four conditions: note that in fMRI, unlike in PET, the zero line does not distinguish absolutely positive from negative effects). In fact, the right IPL had also been found in the conjunction analysis of all five conditions, which can be taken as indication that that was indeed the case. Yet, this effect was most pronounced in the Landolt paradigm. It might be that the nature of the stimuli had an influence here: in the Landolt condition, subjects made more misses than false alarms. A similar pattern was observed for the PW condition, i.e., that of the four orthographic conditions in which neither semantic nor syntactic information would influence or guide reading behavior. For the Landolt condition, the similarity between targets and standard rings might have additionally affected the brain systems involved in the task.

### Semantic and syntactic processing

The lexical-semantic and syntactic contrasts were computed as functional localizers in order to see whether Landolt reading involved any of these processes. Left-hemispheric brain activation was expected for the semantic and syntactic contrasts, especially in the frontal and temporal lobes. Vigneau et al. (2006) ascribe the analysis of both semantic and syntactic information to these anatomical structures. Moreover, Whitney et al. (2011) talk of a semantic network, which is anchored, in the left inferior frontal gyrus and in the posterior part of the middle temporal gyrus, an area that seems to be very important in semantic processing. For syntactic processing, many studies have identified the left inferior frontal cortex as a pivotal region (e.g., Friederici et al., 2000a,b; Santi and Grodzinsky, 2010; Makuuchi et al., 2013) that may interact closely with the left anterior temporal lobe.

The present results are very much in line with this pattern of data. There was a clear effect for the syntax contrast in the left inferior frontal and anterior temporal cortex, which was distinct from the semantic effect in the pMTG. For semantic processing, no left inferior component was observed. This was probably the case because in the present paradigm no selection was required (cf. Thompson-Schill et al., 1997; Heim et al., 2009; for the discussion about the role of pMTG in semantic representation vs. selection cf. Whitney et al., 2011, 2012). Most importantly, as predicted, none of these regions were implicated in Landolt reading, neither in the baseline contrast nor in the conjunction identifying the unique activation for Landolt reading over all other conditions.

## Conclusions

The present study showed that “reading without words” does not lead to significant activation in language processing areas but rather recruits right hemispheric areas in the parietal lobe to a higher degree than the four orthographic conditions. These right-hemispheric areas are involved in both attentional processes and gaze orienting. In combination with earlier behavioral and eye tracking studies, the present data indicate that the Landolt paradigm might be used in future studies to investigate reading in the absence of lexical, syntactic, or phonological-orthographic influences. One application could be in dyslexic reading, which can result from different kinds of underlying deficits. In addition to (or instead of) frequently encountered phonological difficulties (e.g., Ramus et al., 2003), some dyslexic readers show profound difficulties in visual attention (Valdois et al., 2003; Bosse et al., 2007; Heim et al., 2008). First investigations whether, and if so in how far, dyslexic children show uncommon gaze patterns while reading non-lexical material, suggest that a subgroup of children with dyslexia has difficulties in gaze patterns in the Landolt paradigm (Günther et al., 2013; for earlier results on a discrepancy between eye movements during visual search vs. reading in dyslexia cf. Prado et al., 2007). The Landolt paradigm might prove to be a novel tool to tap into these processes relevant for successful reading without interference from pre-lexical information in order to isolate such visuo-attentional processes and the underlying neurofunctional components.

### Conflict of interest statement

The authors declare that the research was conducted in the absence of any commercial or financial relationships that could be construed as a potential conflict of interest.

## Footnotes

^1^Note that this preliminary work at present consists to a large extent of published abstracts and academic theses.
